# Commencements

**Published:** 1895-09

**Authors:** 


### COMMENCEMENTS.

### Detroit College of Medicine—Dental Department.

The second annual commencement exercises of the Department of Dental Surgery of the Detroit College of Medicine were held at the Strassburg Academy, Detroit, Mich., on Monday evening, June 17, 1895.

Addresses were delivered by Professor T. A. McGraw, M.D., J. Ward House, D.D.S., Professor George L. Field, D.D.S., and Professor J. Taft.

The number of matriculates for the session was eighty-one.

The degree of D.D.S. was conferred on the following graduates by Hon. Sidney D. Miller, president of the college:

| NAME. | RESIDENCE. | NAME. | RESIDENCE. |
|---|---|---|---|
| H. R. Barclay | Ontario | L. C. Moore | Michigan |
| A. D. Buchanan | Ontario | E. McDonald | Ontario |
| T. J. Collins | Ontario | J. W. McQueen | Ontario |
| W. E. Cowen | Ontario | H. Nesbit | Ontario |
| J. H. Dunn | Michigan | S. C. Smith | Michigan |
| M. A. Gardner | Michigan | L. N. Wigle | Ontario |
| W. G. Heasley | Michigan | G. A. Wilson | Ontario |
| J. F. Mackenzie | Ontario | C. P. Wood | Michigan |
| A. J. Monagin | Michigan | F. W. Young | Michigan |

### University of Minnesota—Dental Department.

The annual commencement exercises of the Dental Department of the University of Minnesota were held, in connection with those of the law, medical and other departments of the university, at the Exposition Building, Minneapolis, Minn., June 6, 1895.

The number of matriculates for the session was seventy-three.

The degree of D.M.D. was conferred on the following graduates by Cyrus Northrup, LL.D., president of the university:

| NAME. | RESIDENCE. | NAME. | RESIDENCE. |
|---|---|---|---|
| Henry C. Babcock | Minnesota | Frank S. Robinson | Minnesota |
| Frederick E. Cobb | Minnesota | Arthur J. Sauer | Minnesota |
| William A. Demo | Minnesota | Erwin L. Sinclair | Minnesota |
| Herbert B. Hurd | Minnesota | George S. Todd | Wisconsin |
| Frank H. Kyle | Minnesota | Frank J. Wagner | Minnesota |
| Mark O. Nelson | Minnesota | Nathan L. Watson | Minnesota |

### American College of Dental Surgery.

The ninth annual commencement exercises of the American College of Dental Surgery were held at the Grand Opera House, Chicago, Ills., on Thursday, April 4, 1895, at 3:30 P. M.

The number of matriculates for the session was three hundred and fourteen.

The degree of D.D.S. was conferred on the following graduates by Professor B. J. Cigrand, B.S., D.D.S., president of the board of directors:

| NAME. | RESIDENCE. | NAME. | RESIDENBE. |
|---|---|---|---|
| Florence R. Atkinson | Illinois | Edward F. Kenyon | Illinois |
| Michael L. Aren | Illinois | William Knedendorf | Illinois |
| John Bradbury | Illinois | Emil Kark | Wisconsin |
| Harry G. Burke | New York | Maurice Kraus, M.D | Austria |
| Frank W. Booth | Iowa | Samuel Lutz | Illinois |
| Charles Norman | Iowa | William H. Lind | Wisconsin |
| Robert B. Bogle; M.D | Tennessee | Chauncey C. Landon | Indiana |
| John B. Buehner | Illinois | John M. Little | Pennsylvania |
| Frederick P. Bushspies | Illinois | Leroy Lewis, B.Sc | Oregon |
| Frank Bisewski | Illinois | Martin L. Menges | Indiana |
| Francis N. Brown | Illinois | James W. Murray | Iowa |
| A. A. Clinkenbeard | Illinois | William G. McDavitt | Illinois |
| Louis P. Coleman | Iowa | Chas. F. P. O'Connor | Illinois |
| George B. Crissman | Illinois | Waldo A. Overholser | Illinois |
| Arthur Enders | Michigan | Charles B. Payton | Iowa |
| Frank T. Edwards | N. Dakota | Gideon A. Price | Illinois |
| Augustine A. Flick | Pennsylvania | James S. Reed | Nebraska |
| Charles L. Good | Minnesota | R. F. Rowdybush | Illinois |
| Jasper E. Green | Iowa | Meredith Rice | Illinois |
| Ira C. Gamern | Illinois | Elmer B. Roberts | Iowa |
| Frank E. Gunther | Illinois | James L. Russell | Iowa |
| William M. Griffith | Illinois | Frederick C. Rieck | Pennsylvania |
| William G. Hay | Wisconsin | Theo. G. Scholz | Illinois |
| Harry G. Hitchcock | Wisconsin | Nathan W. Smith | Ohio |
| Harry D. Haffa | Iowa | Harry P. Stewart | Ohio |
| Hans M. Heggland | Illinois | Nelson I. Sims | England |
| John H. Hartinger | Indiana | Frank H. Skinner | Illinois |
| Peter Hall | Canada | Andrew C. Thompson | Illinois |
| Walter C. Hunt | Washington | Frank R. Timmermann | Illinois |
| Clinton L. Hopkins | Texas | Albert P. Voorhies | Louisiana |
| Edson B. Jacobs | Ohio | Casimer Wolpers | Ohio |
| Oscar A. Johnson | Illinois | Isaac Wiener | Mississippi |
| Edward A. Kelly | Illinois | George R. White | Illinois |

### University of Pennsylvania—Department of Dentistry.

At a public commencement, held Thursday, June, 13, 1895, at the American Academy of Music, Philadelphia, Pa., the degree of Doctor of Dental Surgery was conferred by Charles C. Harrison, A.M., provost, upon the following candidates, after which an address was delivered by Horatio C. Wood, M.D., L.L.D.,

Professor of Materia Medica, Pharmacy, and General Therapeutics, and Clinical Professor of Nervous Diseases:

| NAME. | RESIDENCE. |
|---|---|
| William C. Achard | Switzerland |
| William L. Aitken | Australia |
| George S. Allen | Illinois |
| William P. Angle | Pennsylvania |
| Carlos A. Barrios | Nicaragua |
| Hass D. Best | Pennsylvania |
| A. Mark Bradner | Pennsylvania |
| Charles A. Bushong | Pennsylvania |
| D. R Campbell, L.D.S. | Scotland |
| Victor Carballo | Uruguay |
| Harry L. Cleaver | Pennsylvania |
| Walter E. Decker | Pennsylvania |
| W. E. S. Dobbyn | Australia |
| Edmond J. Donnegan | Pennsylvania |
| William Dudderidge | Canada |
| Jonas [illegible]. Dudley | Massachusetts |
| David Dunlop, L.D.S. | Scotland |
| Charles S. Evans | Pennsylvania |
| Joseph E. Faulk | Pennsylvania |
| J. D. Forrest, L.D.S. | Scotland |
| Walter R. Garretson | Iowa |
| Willis P. Grandy | Arkansas |
| Sheward Hagerty | Pennsylvania |
| George H. Hahn | Pennsylvania |
| William E. Harris | Virginia |
| Newton C. Hassell | Pennsylvania |
| Edward A. Hoenig | Pennsylvania |
| J. Herbert Hood | Pennsylvania |
| Joseph M. Houston | Pennsylvania |
| C. S. Hurlbut, Jr. | Massachusetts |
| Fred C. Kemble | Ohio |
| John A. Kilmore | Pennsylvania |
| Franklin F. Kribbs | Pennsylvania |
| Oscar Lang | Wisconsin |
| Roman J. Levy | Russia |
| S. A. A. Lewis | Rhode Island |
| J. A. Lopez, Lascano | Ecuador |
| Jose Rafael Madan | Cuba |
| J. Boyd Mader | Pennsylvania |
| Arthur G. Maitland | New Zealand |
| F. B. Manchester | New Jersey |
| R. Middleton, D.V.S. | Pennsylvania |
| W. J. Moorhead | Pennsylvania |
| Charles S. Myers | Pennsylvania |
| Samuel F. Nabers | Alabama |
| William B. Noble | Pennsylvania |
| Charles F. Odell | Ohio |
| Alfredo Pimienta | Spain |
| Frank J. Potter | New York |
| Wm. T. Robinson | Pennsylvania |
| Armand Rous | Indiana |
| G. B. Saxenmeyer | Pennsylvania |
| J. Harry Sehaffer | New York |
| A. O. Schwabe | Austria-Hungary |
| Daniel E. Sorg | Pennsylvania |
| Adam C. Spangler | Pennsylvania |
| Rolof B. Stanley | Pennsylvania |
| Ira B. Stilson | Connecticut |
| H. C. Sturtevant | Pennsylvania |
| Isaac R. Tann | Pennsylvania |
| Fred G. Taylor | Wisconsin |
| Philip A. Traynor | Delaware |
| Clyde A. Van Valin | Pennsylvania |
| David S. Watson | Illinois |
| T. Fred'k Watters | Ohio |
| Frank P. Welch | Oregon |
| Frederick M. Wells | Canada |
| William C. Wells | New York |
| Edward Wishart | Pennsylvania |
| William H. Yale | California |

The number of matriculates for the session was two hundred and seventy-eight.

### Northwestern College of Dental Surgery.

The tenth annual commencement exercises of the Northwestern College of Dental Surgery were held at Kimball Rehearsal Hall, Chicago, Ill., on Wednesday, April 10, 1895.

The valedictory address was delivered by James Archibald Black, B.S., and the doctorate address by Professor F. H. B. McDowell.

The number of matriculates for the session was forty-three.

The degree of D.D.S., was conferred on the following graduates by Dr. J. A. Whipple, dean of the faculty: M. O. Reed, Illinois; W. P. Ranger, Illinois; J. A. Black, Ontario, Canada; J. C. Packard, Wisconsin; Anton Mueller, Austria.

### University of California—College of Dentistry.

The annual commencement exercises of the College of Dentistry of the University of California were held at Odd Fellows' Hall, San Francisco, Cal., on Thursday evening, June 13, 1895.

An address on behalf of the faculty was delivered by William Cormack Reith, D.D.S.

The number of matriculates for the session was one hundred and seventy.

The degree of D.D.S. was conferred on the following graduates by Martin kellogg, A.M.. L.L.D., president of the university:

William A. Atwood.

Federick R. Axton.

Thomas F. Barrett, B.S.

Herbert A. Bernard.

John N. Borger.

John B. Bowles.

James A. Brown.

Louis E. Brun, B.S.

Byron L. Carpenter.

Ralph C. Coleman.

David M. Coney.

Eugene M. Dodson.

Oscar P. Fitch.

James G. Fitzgibbon.

Arthur M. Flood.

Arthur J. Ford.

Reuben L. Hale.

Eminel P. Halsted.

John R. Hardy.

Charles P. Hauselt.

Edward S. Holloway.

Joseph A. Jeffrey.

William B. Ludlow, Jr.

Francis A. McCan.

Amiel Morris.

Robert E. O'Connell.

Clarence H. Pearce.

Robert H. Porterfield.

Frederick E. Sawyer.

Leo Sichel.

Walter E. Singleton.

Robert W. Smith.

Harley H. Stephenson.

Edward L. Strain.

Arthur L. Tibbetts.

Newton B. Wachhorst.

Edwin R. Waterman.

Frederick H. White.

Total, 38,

